# Psychological Distress and Its Association With Quality of Life in Organ Transplant Recipients During COVID-19 Pandemic

**DOI:** 10.3389/fpsyt.2021.690295

**Published:** 2021-06-24

**Authors:** Zhongxiang Cai, Xin Cai, Yujuan Song, Dianzhen Wang, Yanbing Zhang, Simeng Ma, Shiming Tang, Hanping Bai, Huawei Tan, Ruiting Li, Lihua Yao, Zhongchun Liu, Gaohua Wang, Ying Wang

**Affiliations:** ^1^Nursing Office, Renmin Hospital of Wuhan University, Wuhan, China; ^2^Department of Cardiovascular Surgery, Renmin Hospital of Wuhan University, Wuhan, China; ^3^The Nephrology Division and Dialysis Transplant Center, Renmin Hospital of Wuhan University, Wuhan, China; ^4^Department of Hepatobiliary Surgery, Renmin Hospital of Wuhan University, Wuhan, China; ^5^Department of Psychiatry, Renmin Hospital of Wuhan University, Wuhan, China

**Keywords:** coronavirus disease, organ transport, psychological distress, quality of life, cross-sectional study

## Abstract

**Objectives:** The coronavirus disease 2019 (COVID-19) pandemic may have an impact on the psychological distress of organ transplant recipients. We aimed to assess the status of psychological distress and its association with quality of life (QoL) in organ transplant recipients during the COVID-19 pandemic.

**Materials and Methods:** A cross-sectional survey was carried out with 305 organ transplant recipients during March 30 and April 2, 2020, in Wuhan. Psychological distress comprised depression, anxiety, insomnia, and post-traumatic stress disorder (PTSD), which were assessed using the Patient Health Questionnaire-9, the seven-item Generalized Anxiety Disorder questionnaire, the Insomnia Severity Index, and Impact of event scale-revised. QoL was assessed using the Chinese version of the short Form 36-item health survey.

**Results:** The prevalence of depression, anxiety, insomnia, and PTSD in organ transplant recipients was 13.4, 6.9, 11.8, and 30.5%, respectively. Organ transplant recipients with depression had significantly lower scores in all eight dimensions of QoL compared with participants without depression (all *p* < 0.05). Lower scores on the QoL dimensions of role physical, bodily pain, general health, vitality, role emotional, and mental health were found in organ transplant recipients with anxiety, insomnia, or PTSD compared with their counterparts without the respective disorder (all *p* < 0.05).

**Limitation:** The cross-sectional study design limited us to make causal conclusion and the influence of potential confounders cannot be ruled out.

**Conclusions:** Psychological distress was prevalent in organ transplant recipients during the COVID-19 pandemic, and those with depression, anxiety, insomnia, and PTSD had poorer QoL. Therefore, timely psychological counseling, COVID-19 related health education, and essential community medical services should be provided to organ transplant recipients to relieve their psychological distress, and to improve their QoL.

## Introduction

The outbreak of coronavirus disease 2019 (COVID-19), first reported in China (1), has become a pandemic. As of November 22, 2020, more than 57.8 million confirmed cases and 1.3 million deaths had been reported worldwide (2). During the COVID-19 pandemic, the psychological distress among the population can be prominent because of social isolation, uncertainty of the future, fear of being infected, and overwhelming negative news portrayal in mass media coverage (3, 4). The mechanisms that people get into psychological distress are implicated, for instance, Serafini et al. found that extreme sensory processing patterns show a complex association with depression, and impulsivity, alexithymia, and hopelessness (5). Numerous studies have proven that both healthcare workers and the general public were associated high psychological burden during this crisis (6). It was suggested that psychological interventions targeting high-risk populations with heavy psychological distress are in urgent need, and the importance of protective factors including sufficient medical resources, up-to-date and accurate information, and precautionary measures should be stressed (6, 7).

End-stage organ failure is a serious condition associated with an increased risk of mortality (8, 9). Organ transplantation is often the only treatment for patients with end-stage organ failure. The number of patients undergoing organ transplantation is increasing (10), with more than 100,000 organ transplantations performed annually worldwide (11), and a post-transplant survival exceeding 85-90% in the first year and 70-75% at 5 years (12). Kidney transplantation is the most frequent, globally, followed by liver and heart transplantations. Patients with end-stage organ failure suffer from severe physical and psychological symptoms (13). QoL is a major index to the evaluate the efficacy of medical intervention among patients with end-stage organ failure. Organ transplantation has been demonstrated to be the best treatment both for quality of life (QoL) and cost effectiveness (14–16).

Organ transplant recipients are patients who have received organ transplantation. A large proportion of organ transplant recipients suffer psychological distress, including symptoms of depression (17), anxiety (18), and post-traumatic stress disorder (PTSD) (11). During the COVID-19 pandemic, moderate to severe depression and anxiety were demonstrated to be independently associated with increased risk of low QoL among healthcare workers (19). Affected by the COVID-19 pandemic, organ transplant recipients may suffer more apparent psychological distress than before, and as a result may endure worsen QoL. Nevertheless, the status of psychological distress during the COVID-19 pandemic and its association with QoL in organ transplant recipients is unknown. Therefore, this study involved a cross-sectional survey with organ transplant recipients to assess their level of psychological distress and QoL, and explore the association between the two, during the COVID-19 pandemic.

## Materials and Methods

### Study Participants

This cross-sectional study was conducted between March 30 and April 2, 2020. It was approved by the Clinical Research Ethics Committee of Renmin Hospital of Wuhan University (WDRY2020-K004). Organ transplant recipients were identified from the medical records of Renmin Hospital of Wuhan University. Individuals were included if they underwent kidney, heart, or liver transplantation from January 2015 to December 2019, and were excluded if they died, could not complete the questionnaires, or refused to sign the informed consent. 305 from 342 invited organ transplant recipients (with a response rate of 89.2%) agreed to participate in this study and signed the informed consent before their participation. In the informed consent forms, participants were encouraged to seek psychological assistance through a free online psychological support system, in which psychologist and psychiatrist could provide online psychological assistance (20).

### Assessment of Psychological Distress

As described in our previous studies (4, 21), symptoms of depression, anxiety, insomnia, and PTSD were assessed using the Patient Health Questionnaire-9 (PHQ-9) (22), 7-item Generalized Anxiety Disorder (GAD-7) questionnaire (23), Insomnia Severity Index (ISI) (24), and Impact of Event Scale-Revised (IES-R) (24), respectively. The cut-off scores for identifying major depression, anxiety, insomnia, and PTSD are 10, 10, 15, and 22, on the respective scales. The validity and reliability of these instruments have been confirmed in Chinese population (25–28).

### Assessment of QoL

QoL was evaluated using the Chinese version of the Short Form 36-Item Health Survey (SF-36) (29). The SF-36 comprises 36 items that cover eight dimensions: physical functioning (PF), role physical (RP), bodily pain (BP), general health (GH), vitality (VT), social functioning (SF), role emotional (RE), and mental health (MH). The score for each dimension ranges from 0 to 100, with higher values indicating better functioning and fewer limitations.

### Assessment of Covariates

Covariates were collected by questionnaires, including age, gender, marital status, education level, living location, living condition, type of organ transplantation, post-operative time, comorbidities, and worry about infection.

### Statistical Analysis

Data analysis was conducted using SPSS version 20.0 (IBM Corp., Armonk, New York, United States). The associations between sociodemographic variables, psychological distress, and QoL were analyzed by the analysis of variance (ANOVA) or the Wilcoxon rank sum test where appropriate. The significance level was set as α = 0.05.

## Results

### Characteristics of Organ Transplant Recipients

Table 1 presents the characteristics of the organ transplant recipients in this study. Participants were 196 males and 109 females, with a mean (SD) age of 43.1 (10.7) years. A total of 248 (81.3%) were kidney transplant recipients, 39 (12.8) were heart transplant recipients, and 18 (5.9%) were liver transplant recipients. The median post-operative time was 17.1 months. The participants tended to be of Han ethnic group (89.5%), be married (81.3%), have an educational level of less than undergraduate (53.1%), live in an urban area (76.4%), live with others (95.7%), worry about infection (70.2%), and have comorbidities (57.7%).

**Table 1:** Characteristics of organ transplant recipients.

**Variables**	**No. of participants (%)**
Total	305 (100.0)
Age (year), IQR	44 (35,50)
**Gender**	
Male	196 (64.3)
Female	109 (35.7)
**Ethnic groups**	
Han ethnic group	273 (89.5)
Ethnic minorities	32 (10.5)
**Marital status**	
Unmarried	57 (18.7)
Married^*^	248(81.3)
**Education level**	
Lower than undergraduate	162 (53.1)
Undergraduate or higher	143 (46.9)
**Living area**	
Unban area	233 (76.4)
Rural area	72 (23.6)
**Living status**	
Alone	13 (4.3)
With others	292 (95.7)
**Worry about infection**	
Yes	214 (70.2)
No	91 (29.8)
**Type of transplantation**	
Kidney	248 (81.3)
Heart	39 (12.8)
Liver	18 (5.9)
Postoperative time (month), IQR	13 (8,21)
**Comorbidities**
Yes	176 (57.7)
No	129 (42.3)

*IQR, interquartile range*.

* *Including 13 widowed or divorced participants*.

### Psychological Distress

Table 2 presents the status of psychological distress in organ transplant recipients during the COVID-19 pandemic. The estimated prevalence of depression, anxiety, insomnia, and PTSD was 13.4, 6.9, 11.8, and 30.5%, respectively. Older organ transplant recipients had a higher prevalence of insomnia than younger ones. Participants with comorbidities had a higher prevalence of insomnia than those without. A higher prevalence of PTSD was observed in those of older age, married, having an education level lower than undergraduate, and being worried about infection compared to their respective counterparts.

**Table 2:** Psychological distress in organ transplant recipients during COVID-19 pandemic.

**Variables**	**Depression**			**Anxiety**			**Insomnia**			**PTSD**		
	**Yes**	**No**	***P***	**Yes**	**No**	***P***	**Yes**	**No**	***P***	**Yes**	**No**	***P***
Total	41 (13.4)	264 (86.6)		21 (6.9)	284 (93.1)		36 (11.8)	269 (88.2)		93 (30.5)	212 (69.5)	
**Gender**												
Male	28 (14.3)	168 (85.7)	0.563	14 (7.1)	182 (92.9)	0.812	24 (12.2)	172 (87.8)	0.749	56 (28.6)	140 (71.4)	0.329
Female	13 (11.9)	96 (88.1)		7 (6.4)	102 (93.6)		12 (11.0)	97 (89.0)		37 (33.9)	72 (66.1)	
**Age (year)**												
<30	2 (6.7)	28 (93.3)	0.298	2 (6.7)	28 (93.3)	0.335	1 (3.3)	29 (96.7)	0.003	5 (16.7)	25 (83.3)	0.002
30-	11 (12.0)	81 (88.0)		4 (4.3)	88 (95.7)		6 (6.5)	86 (93.5)		19 (20.7)	73 (79.3)	
40-	21 (17.8)	97 (82.2)		12 (10.2)	106 (89.8)		24 (20.3)	94 (79.7)		50 (42.4)	68 (57.6)	
≥50	7 (10.8)	58 (89.2)		3 (4.6)	62 (95.4)		5 (7.7)	60 (92.3)		19 (29.2)	46 (70.8)	
**Marital status**												
Unmarried	8 (14.0)	49 (86.0)	0.884	5 (8.8)	52 (91.2)	0.562	3 (5.3)	54 (94.7)	0.090	9 (15.8)	48 (84.2)	0.007
Married^*^	33 (13.3)	215 (86.7)		16 (6.5)	232 (93.5)		33 (13.3)	215 (86.7)		84 (33.9)	164 (66.1)	
**Education level**												
Lower than undergraduate	26 (16.0)	136 (84.0)	0.155	14 (8.6)	148 (91.4)	0.197	15 (9.3)	147 (90.7)	0.143	58 (35.8)	104 (64.2)	0.032
Undergraduate or higher	15 (10.5)	128 (89.5)		7 (4.9)	136 (95.1)		21 (14.7)	122 (85.3)		35 (24.5)	108 (75.5)	
**Ethnic groups**												
Han Ethnic group	39 (14.3)	234 (85.7)	0.279	21 (7.7)	252 (92.3)	0.145	31 (11.4)	242 (88.6)	0.559	83 (30.4)	190 (69.6)	0.922
Ethnic minorities	2 (6.3)	30 (93.8)		0 (0.0)	32 (100.0)		5 (15.6)	27 (84.4)		10 (31.3)	22 (68.8)	
**Living area**												
Unban area	33 (14.2)	200 (85.8)	0.507	16 (6.9)	217 (93.1)	1.000	32 (13.7)	201 (86.3)	0.060	70 (30.0)	163 (70.0)	0.759
Rural area	8 (11.1)	64 (88.9)		5 (6.9)	67 (93.1)		4 (5.6)	68 (94.4)		23 (31.9)	49 (68.1)	
**Living status**												
Alone	4 (30.8)	9 (69.2)	0.081	1 (7.7)	12 (92.3)	1.000	2 (15.4)	11 (84.6)	0.657	7 (53.8)	6 (46.2)	0.071
With others	37 (12.7)	255 (87.3)		20 (6.8)	272 (93.2)		34 (11.6)	258 (88.4)		86 (29.5)	206 (70.5)	
**Worry about infection**												
Yes	28 (13.1)	186 (86.9)	0.778	15 (7.0)	199 (93.0)	0.896	25 (11.7)	189 (88.3)	0.920	78 (36.4)	136 (63.6)	0.001
No	13 (14.3)	78 (85.7)		6 (6.6)	85 (93.4)		11 (12.1)	80 (87.9)		15 (16.5)	76 (83.5)	
**Type of transplantation**												
Kidney	6 (15.4)	33 (84.6)	0.470	6 (15.4)	33 (84.6)	0.134	7 (17.9)	32 (82.1)	0.056	13 (33.3)	26 (66.7)	0.359
Heart	31 (12.5)	217 (87.5)		14 (5.6)	234 (94.4)		28 (11.3)	220 (88.3)		72 (29.0)	176 (71.0)	
Liver	4 (22.2)	14 (77.8)		1 (5.6)	17 (94.4)		1 (5.6)	18 (94.4)		8 (44.4)	10 (55.6)	
**Post-operative time (month)**												
<12	21 (14.8)	121 (85.2)	0.520	8 (5.6)	134 (94.4)	0.420	18 (12.7)	124 (87.3)	0.659	38 (26.8)	104 (73.2)	0.186
≥12	20 (12.3)	143 (87.7)		13 (8.0)	150 (92.0)		18 (11.0)	145 (89.0)		55 (33.7)	108 (66.3)	
**Comorbidities**												
Yes	29 (16.5)	147 (83.5)	0.070	16 (9.1)	160 (90.9)	0.076	29 (16.5)	147 (83.5)	0.003	58 (33.0)	118 (67.0)	0.275
No	12 (9.3)	117 (90.7)		5 (3.9)	124 (96.1)		7 (5.4)	122 (94.6)		35 (27.1)	94 (72.9)	

*PTSD, post-traumatic stress disorder*.

* *Including 13 widowed or divorced participants*.

### QoL

Table 3 displays the status of QoL in organ transplant recipients during the COVID-19 pandemic. The mean scores (standard deviation) for the eight SF-36 dimensions (PF, RP, BP, GH, VT, SF, RE, and MH) were 81.9 (14.0), 55.7 (41.9), 83.8 (16.3), 66.0 (19.6), 73.1 (18.9), 57.3 (17.7), 68.0 (39.3), and 72.8 (18.7). Males had higher PF scores compared with females. Younger participants had higher PF and SF scores than older ones. Unmarried participants had higher PF scores and lower VT scores than their counterparts. Those worried about infection had lower scores on PF, RP, GH, SF, and RE than those who were not worried. Participants who had undergone organ transplantation within 1 year had lower scores on PF and RP compared with those who had undergone transplantation more than 1 year before. Lower GH, VT, and RE scores were observed in participants with comorbidities compared with those without.

**Table 3:** Quality of life in organ transplant recipients during COVID-19 pandemic.

**Variables**	**PF**		**RP**		**BP**		**GH**		**VT**		**SF**		**RE**		**MH**	
	**Score**	**P**	**Score**	**p**	**Score**	**p**	**Score**	**p**	**Score**	**p**	**Score**	**p**	**Score**	**p**	**Score**	**p**
Total	81.9 (14.0)		55.7 (41.9)		83.8 (16.3)		66.0 (19.6)		73.1 (18.9)		57.3 (17.7)		68.0 (39.3)		72.8 (18.7)	
**Gender**																
Male	83.4 (13.5)	0.003	56.0 (41.8)	0.853	83.9 (15.1)	0.803	65.6 (19.8)	0.772	73.9 (18.5)	0.390	57.1 (16.6)	0.592	69.6 (39.0)	0.251	72.6 (18.8)	0.777
Female	79.3 (14.4)		55.3 (42.1)		83.5 (18.2)		66.7 (19.3)		71.7 (19.6)		57.6 (19.5)		65.1 (39.6)		73.0 (18.6)	
**Age (year)**																
<30	85.0 (11.3)	0.019	55.8 (41.4)	0.588	82.5 (20.5)	0.758	72.6 (18.0)	0.257	69.3 (16.7)	0.184	67.1 (13.3)	0.009	64.4 (41.0)	0.859	73.5 (19.4)	0.172
30-	84.8 (12.1)		57.3 (41.2)		86.0 (13.4)		64.6 (19.6)		72.2 (19.2)		56.5 (18.9)		67.8 (39.4)		70.3 (17.9)	
40-	81.1 (13.3)		52.3 (43.1)		82.9 (16.2)		64.9 (19.6)		73.1 (18.7)		55.2 (16.2)		68.1 (40.0)		73.0 (18.1)	
≥50	77.9 (17.4)		59.6 (39.7)		82.9 (18.1)		66.9 (20.1)		76.1 (19.6)		57.5 (19.1)		69.7 (37.6)		75.5 (20.3)	
**Marital status**																
Unmarried	84.5 (14.0)	0.045	53.1 (41.7)	0.705	83.8 (17.3)	0.951	65.3 (21.5)	0.935	68.7 (18.6)	0.030	59.4 (17.7)	0.751	64.9 (38.5)	0.372	69.8 (19.1)	0.159
Married^*^	81.3 (13.9)		56.4 (41.9)		83.9 (16.1)		66.1 (19.2)		74.1 (18.8)		56.8 (17.7)		68.7 (39.5)		73.4 (18.6)	
**Education level**																
Lower than undergraduate	80.1 (15.0)	0.230	52.3 (42.9)	0.127	83.4 (17.4)	0.971	66.2 (20.0)	0.880	71.2 (19.4)	0.054	56.6 (16.4)	0.211	64.0 (39.7)	0.056	71.2 (19.2)	0.118
Undergraduate or higher	83.2 (12.7)		59.6 (40.5)		84.2 (15.1)		65.7 (19.2)		75.2 (18.1)		58.0 (19.0)		72.5 (38.4)		74.6 (18.1)	
**Ethnic groups**																
Han Ethnic group	81.7 (14.2)	0.501	54.2 (42.0)	0.069	83.9 (15.9)	0.848	65.0 (19.8)	0.012	72.5 (18.9)	0.134	57.4 (18.0)	0.336	66.5 (39.6)	0.055	72.7 (19.0)	0.930
Ethnic minorities	83.9 (11.5)		68.8 (38.6)		83.1 (19.8)		74.7 (15.9)		77.8 (18.0)		55.9 (14.9)		80.2 (34.8)		73.3 (16.3)	
**Living area**																
Unban area	81.7 (13.5)	0.338	56.9 (41.5)	0.420	84.5 (16.0)	0.172	66.2 (20.1)	0.773	74.1 (19.0)	0.039	57.3 (17.7)	0.684	69.7 (38.7)	0.189	73.6 (18.7)	0.079
Rural area	82.4 (15.5)		52.1 (42.9)		81.6 (17.2)		65.4 (18.1)		69.9 (18.4)		57.1 (17.9)		62.5 (40.7)		69.9 (18.6)	
**Living status**																
Alone	85.4 (12.8)	0.350	67.3 (40.0)	0.336	74.5 (27.8)	0.151	61.2 (24.7)	0.314	64.2 (25.9)	0.228	55.8 (20.8)	0.426	64.1 (46.1)	0.815	66.8 (19.1)	0.206
With others	81.8 (14.0)		55.2 (41.9)		84.2 (15.5)		66.2 (19.4)		73.5 (18.5)		57.3 (17.6)		68.2 (39.0)		73.0 (18.7)	
**Worry about infection**																
Yes	80.8 (13.9)	0.013	51.3 (41.6)	0.002	83.5 (16.5)	0.520	63.9 (19.0)	0.002	72.6 (18.4)	0.332	55.6 (18.3)	0.015	63.4 (40.8)	0.003	72.2 (18.2)	0.332
No	84.4 (13.9)		66.2 (40.9)		84.5 (15.9)		71.0 (20.3)		74.2 (19.9)		61.1 (15.4)		78.8 (33.2)		74.1 (19.9)	
**Type of transplantation**																
Kidney	74.1 (21.6)	0.047	53.9 (43.9)	0.186	80.4 (20.7)	0.681	61.2 (21.5)	0.196	68.6 (23.3)	0.490	59.0 (18.4)	0.779	65.0 (40.4)	0.733	71.7 (19.9)	0.911
Heart	82.9 (11.9)		54.7 (41.4)		84.4 (15.7)		66.4 (19.3)		73.6 (18.1)		57.1 (17.7)		68.0 (39.2)		73.1 (18.4)	
Liver	84.4 (14.8)		73.6 (41.5)		82.6 (13.9)		70.1 (19.1)		75.6 (18.4)		56.3 (16.7)		74.1 (38.9)		71.1 (20.8)	
**Post-operative time (month)**																
<12	79.1 (14.6)	<0.001	45.6 (42.2)	<0.001	81.7 (17.8)	0.038	65.7 (20.5)	0.909	72.3 (19.8)	0.675	55.8 (20.0)	0.324	65.5 (41.1)	0.471	73.6 (19.6)	0.232
≥12	84.4 (12.9)		64.6 (39.6)		85.6 (14.6)		66.3 (18.9)		73.8 (18.1)		58.5 (15.3)		70.1 (37.5)		72.0 (17.9)	
**Comorbidities**																
Yes	82.2 (13.3)	0.980	53.0 (42.5)	0.191	83.3 (16.3)	0.342	62.1 (19.2)	<0.001	70.5 (18.9)	0.003	56.0 (17.2)	0.159	64.4 (39.8)	0.041	72.0 (18.6)	0.371
No	81.5 (14.9)		59.5 (40.8)		84.5 (16.3)		71.3 (19.0)		76.6 (18.4)		58.9 (18.3)		72.9 (38.1)		73.8 (18.8)	

*PF, physical functioning; RP, role physical; BP, bodily pain; GH, general health; VT, vitality; SF, social functioning; RE, role emotional; MH, mental health*.

* *Including 13 widowed or divorced participants*.

### Association of Psychological Distress With QoL

Table 4 shows the results regarding the association of psychological distress with QoL in organ transplant recipients during the COVID-19 pandemic. Organ transplant recipients with depression had significantly lower scores in all eight dimensions of QoL compared with those without depression (all *p* < 0.05). Lower scores on RP, BP, GH, VT, RE, and MH were found in organ transplant recipients with anxiety, insomnia, or PTSD compared with those without the respective disorder (all *p* < 0.05).

**Table 4:** Association of psychological distress with quality of life in organ transplant recipients during COVID-19 pandemic.

**Variables**	**PF**		**RP**		**BP**		**GH**		**VT**		**SF**		**RE**		**MH**	
	**Score**	**p**	**Score**	**p**	**Score**	**p**	**Score**	**p**	**Score**	**p**	**Score**	**p**	**Score**	**p**	**Score**	**p**
**Depression**																
Yes	74.3 (19.1)	0.005	23.2 (34.2)	<0.001	71.3 (19.2)	<0.001	44.8 (16.9)	<0.001	47.7 (17.9)	<0.001	47.9 (21.8)	<0.001	22.0 (34.6)	<0.001	48.1 (16.6)	<0.001
No	83.1 (12.6)		60.8 (40.7)		85.7 (14.9)		69.3 (17.9)		77.0 (15.7)		58.7 (16.5)		75.1 (34.9)		76.6 (15.9)	
**Anxiety**																
Yes	76.9 (20.4)	0.336	36.9 (38.4)	0.037	75.3 (20.8)	0.022	45.8 (16.5)	<0.001	49.1 (15.9)	<0.001	56.5 (21.0)	0.336	27.0 (37.5)	<0.001	44.2 (15.1)	<0.001
No	82.3 (13.3)		57.1 (41.8)		84.4 (15.8)		67.5 (19.0)		74.9 (17.2)		57.1 (17.4)		71.0 (37.7)		74.9 (17.2)	
**Insomnia**																
Yes	76.5 (18.2)	0.065	37.5 (40.3)	0.008	72.3 (19.4)	<0.001	51.1 (16.4)	<0.001	58.1 (21.7)	<0.001	52.4 (22.5)	0.046	46.3 (44.6)	0.001	56.8 (20.8)	<0.001
No	82.6 (13.2)		58.2 (41.5)		85.3 (15.2)		68.0 (19.2)		75.1 (17.6)		57.9 (16.9)		70.9 (37.6)		74.9 (17.4)	
**PTSD**																
Yes	79.9 (14.2)	0.070	45.4 (41.7)	0.003	81.2 (16.2)	0.032	60.7 (18.3)	0.001	66.0 (19.2)	<0.001	54.0 (16.8)	0.004	50.9 (42.4)	<0.001	62.8 (18.8)	<0.001
No	82.8 (13.8)		60.3 (41.2)		84.9 (16.3)		68.3 (19.7)		76.2 (17.9)		58.7 (17.9)		75.5 (35.3)		77.1 (16.9)	

*PF, physical functioning; RP, role physical; BP, bodily pain; GH, general health; VT, vitality; SF, social functioning; RE, role emotional; MH, mental health; PTSD, post-traumatic stress disorder*.

## Discussion

In this study, the estimated prevalence of depression, anxiety, insomnia, and PTSD in organ transplant recipients was 13.4, 6.9, and 11.8, and 30.5%, respectively. Overall, during the COVID-19 pandemic, organ transplant recipients with psychological distress had poorer QoL than those without psychological distress. More specifically, organ transplant recipients with depression experienced poorer QoL in all the eight dimensions of SF-36 than those without depression, and organ transplant recipients with anxiety, insomnia, and PTSD showed reduced performance in six dimensions of SF-36, namely RP, BP, GH, VT, RE, and MH, relative to their counterparts without the respective disorder. The prevalence of depression and anxiety in organ transplant recipients was in consistent with previous studies, which reported a prevalence of depression ranging from 13 to 37% (17), and a prevalence of anxiety ranging from 3 to 18% (18, 30). The prevalence of PTSD in organ transplant recipients seemed to be higher than that in a prospective cohort study, which found that the prevalence of PTSD in liver transplant recipients before transplantation and at 1-year post-transplantation was 10.5 and 6.3%, respectively (31). It has been suggested that more organ transplant recipients suffered from PTSD during the COVID-19 pandemic compared with normal conditions (31). However, the difference in PTSD prevalence might be partially attributed to variations in study populations, assessment tools, and the time of assessment. When compared with general population, organ transplant recipients had higher prevalence of depression and insomnia, lower prevalence of anxiety, and similar prevalence of PTSD, during the same stage of COVID-19 pandemic in China (32, 33).

Organ transplant recipients have been found to exhibit various forms of psychological distress such as depression, anxiety, and stress associated with physical and psychosocial stress factors such as life-threatening illness, transplant surgery, pain, and intensive care unit stays with mechanical ventilation and possible delirium (11). Although psychological problems may decrease after transplantation as patient outcomes and QoL improve (34), one-third of heart transplant recipients experienced high levels of psychological distress in the year following transplantation (35, 36). Notably, being a public health emergency, the COVID-19 pandemic has had a significant psychological impact on both the general population and healthcare workers. Prior to this study, the status of psychological distress in transplant recipients during the COVID-19 pandemic was unknown. Since this study found that psychological distress is prevalent in organ transplant recipients, we therefore suggest that more attention be paid to the mental health status of this patient population and social support be provided for them during the COVID-19 pandemic.

QoL in organ transplant recipients could be improved post-transportation. Based on a review, in all included studies that used the SF 36 to assess QoL, the PF scores of elderly transplant recipients were significantly higher compared to their age-adjusted norms, while the BP, GH, VT, SF, RE, and MH scores did not differ significantly (37). This has brought hope for organ transplant recipients to reach an acceptable level of QoL after transplantation. However, the QoL in organ transplant recipients could be affected by several factors, such as age, educational level, employment status, family support, and negative emotional states (38). In this study, we provided evidence that symptoms of depression, anxiety, insomnia, and PTSD were associated with poorer QoL in organ transplant recipients. Our findings highlight the importance of implementing essential measures to relieve organ transplant recipients' psychological distress and improve their QoL.

To the best of our knowledge, this is the first study to assess the status of psychological distress and its association with QoL in organ transplant recipients during the COVID-19 pandemic. However, our study had several limitations. We analyzed cross-sectional data from a limited number of participants, and all the variables were self-reported; thus, there is a possibility of recall and misclassification bias, which may lead to misleading results. In addition, our study was conducted during the COVID-19 pandemic, and there was no baseline data that obtained before the outbreak of COVID-19; therefore, we could not assess whether or not the status of psychological distress in organ transplant recipients was more severe during the COVID-19 pandemic. Moreover, the prevalence of psychological distress may decline over time after transplantation; therefore, the present findings should be further confirmed using longitudinal data.

## Conclusions

In conclusion, the current study indicated that psychological distress was prevalent in organ transplant recipients during the COVID-19 pandemic. Moreover, organ transplant recipients with depression, anxiety, insomnia, and PTSD had poorer QoL than their counterparts without the respective disorder. Timely psychological counseling, COVID-19 related health education, and essential community medical services should thus be provided to organ transplant recipients to relieve their psychological distress, and improve their QoL.

## Data Availability Statement

The raw data supporting the conclusions of this article will be made available by the authors, without undue reservation.

## Ethics Statement

The studies involving human participants were reviewed and approved by Clinical Research Ethics Committee of Renmin Hospital of Wuhan University. The patients/participants provided their written informed consent to participate in this study.

## Author Contributions

All authors contributed substantially to the conception and design, reviewed the manuscript, and approved the submitted version of the manuscript.

## Conflict of Interest

The authors declare that the research was conducted in the absence of any commercial or financial relationships that could be construed as a potential conflict of interest.
